# A brief review of chikungunya fever: From molecular virology to countermeasures

**DOI:** 10.1016/j.imj.2025.100231

**Published:** 2025-12-25

**Authors:** Xin Zhang, Xiaoxi Li, Tianjun Jiang, Junliang Fu

**Affiliations:** a Senior Department of Infectious Diseases, Chinese PLA General Hospital, Beijing 100039, China; b Department of Clinical Laboratory, The Fifth Medical Center of Chinese PLA General Hospital, Beijing 100039, China

**Keywords:** Chikungunya fever, Chikungunya virus, Arbovirus, Vaccine, Antiviral therapy

## Abstract

Chikungunya fever (CHIKF), resulting from the chikungunya virus infection, has become a major global health issue in recent years. This review summarizes the epidemiology, virology, pathogenesis, clinical manifestations, diagnosis, vaccine development and treatment strategies of the CHIKF. And by integrating the findings from various studies, an attempt is made to propose future research directions and intervention strategies.

## Graphical abstract

**Figure:**
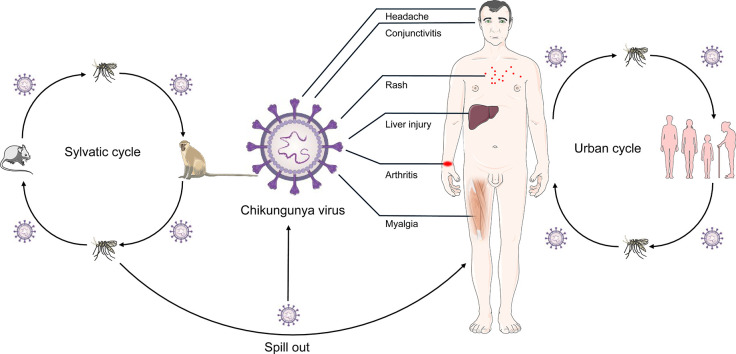

## 1 Introduction

Chikungunya virus (CHIKV), a mosquito-transmitted alphavirus, is the etiological agent of chikungunya fever (CHIKF) and constitutes an increasingly prominent global public health concern. CHIKF is clinically marked by a sudden high fever, maculopapular rash, headache, myalgia, and severe, often debilitating, polyarthralgia. Although the acute phase is generally self-limiting, 30% to over 60% of patients in certain cohorts develop chronic inflammatory rheumatological conditions that may last for months or years. This chronic phase, marked by ongoing joint pain, stiffness, and swelling, presents a major public health issue, leading to considerable declines in quality of life and economic productivity.^1^

The virus mainly spreads to humans via bites from infected female mosquitoes, especially *Aedes aegypti* and *Aedes albopictus*. The *Aedes albopictus*, also known as “Asian tiger mosquito”, with its extensive geographic range and adaptability to cooler climates, has played a crucial role in the global spread of CHIKV beyond tropical regions. Since the early 2000s, CHIKV has undergone a dramatic global resurgence. In the first half of 2025, more than 220,000 cases and 80 fatalities were recorded worldwide across 14 nations, recent local transmission in subtropical urban regions of China, notably in Guangdong Province, reported 16,452 confirmed cases as of 27 September 2025, emphasizes the virus's adaptability to new environments 2, 3. This review offers an in-depth analysis of recent CHIKV research, focusing on its evolving epidemiology and viral evolution, the molecular mechanisms of replication and host-virus interaction, the pathogenesis, advancements in diagnostics, vaccines, and antiviral treatments.

## 2 Epidemiology

### 2.1 Global distribution and outbreak patterns

**Fig. 1:**
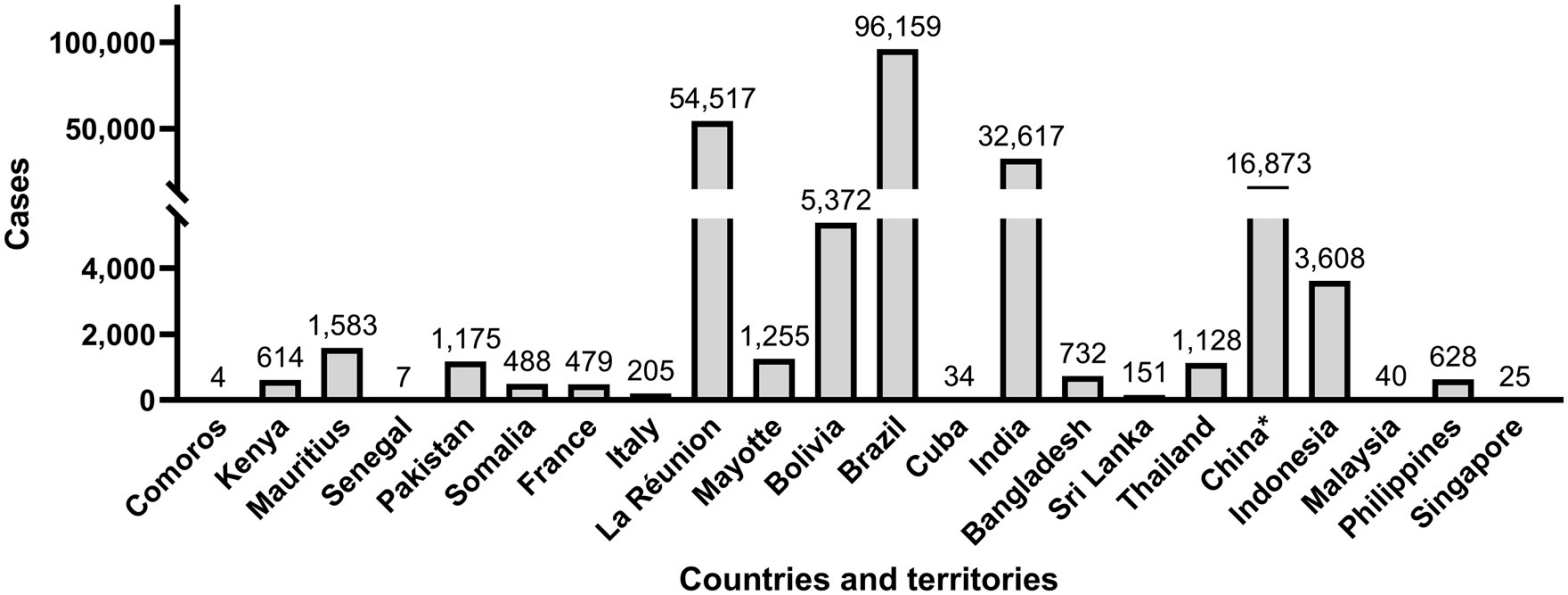
Geographical distribution of CHIKV disease cases, data from January to September 2025.^3^ *: excluding Hong Kong SAR, Macao SAR, and Taiwan, China. *Abbreviations*: SAR, special administrative region; Comoros, Union of the Comoros; Kenya, the Republic of Kenya; Mauritius, the Republic of Mauritius; Senegal, the Republic of Senegal; Pakistan, the Islamic Republic of Pakistan; Somalia, the Federal Republic of Somalia; France, the French Republic; Italy, Repubblica Italiana; Bolivia, Plurinational State of Bolivia; Brazil, the Federative Republic of Brazil; Cuba, the Republic of Cuba; India, the Republic of India; Bangladesh, the People's Republic of Bangladesh; Sri Lanka, the Democratic Socialist Republic of Sri Lanka; Thailand, the Kingdom of Thailand; Indonesia, Republic of Indonesia; Philippines, Republic of the Philippines; Singapore, Republic of Singapore.

Historically, CHIKV was mainly found in Africa and Asia, causing sporadic outbreaks. The global epidemiology of CHIKV has been characterized by its rapid geographic expansion and the occurrence of large-scale, explosive outbreaks over the past two decades (Fig. 1).

In Africa, the continent of origin for CHIKV, the virus continues to circulate, causing both epidemic and endemic disease. A study in the Republic of Senegal has provided evidence for the re-emergence of CHIKV as an endemic, locally sustained transmission cycle, rather than a series of re-introductions from elsewhere.^4^

Asia has also experienced major CHIKV outbreaks. The virus is also being detected in new regions; a study identified the presence of the West African genotype of CHIKV in China, highlighting the risk of viral importation and establishment in non-endemic areas through international travel.^5^ In the People's Republic of Bangladesh (Bangladesh), seroprevalence surveys have been conducted to estimate the true burden of infection in the population, often revealing that a much larger proportion of people have been infected than is captured by official case reports.^6^

In South America, the virus has become firmly established. Investigations in urban settings in the Federative Republic of Brazil (Brazil) have detailed the intense transmission dynamics of the East/Central/South African (ECSA) lineage of CHIKV.^7^ Recent outbreaks, such as a major epidemic in Republic of Paraguay, underscore the virus's capacity for rapid and widespread transmission in immunologically naive populations.^8^ Co-infections between CHIKV and Mayaro virus, O'nyong-nyong virus or Dengue virus have been reported, which can complicate clinical diagnosis and management.^9^, ^10^, ^11^, ^12^, ^13^

### 2.2 Vector ecology and zoonotic cycle

A key driver of CHIKV's global success is its ability to adapt to different mosquito vectors. Specific variants of CHIKV have been shown to affect the transmission competence of *Aedes aegypti*.^14^ At the vector level, the regulation of oxidative stress within the mosquito has been shown to be a key modulator of its antiviral immune response.^15^

The long-term persistence of CHIKV is believed to depend on a sylvatic, or zoonotic, cycle involving non-human hosts. For example, natural infection with CHIKV has been documented in wild lion tamarins (*Leontopithecus* species) in Brazil, confirming their role as hosts in the neotropical sylvatic cycle.^16^ Beyond primates, studies have detected neutralizing antibodies against CHIKV in bats and opossums, suggesting that these mammals may also participate in the virus's maintenance cycle in nature.^17^

### 2.3 Risk factors and population susceptibility

Various risk factors contribute to CHIKV infection and the severity of the disease. Increased risk of severe disease and mortality is associated with older age, male sex, and specific comorbidities like diabetes mellitus, hypertension, and chronic kidney disease.^18^ CHIKV infection in pregnant women is linked to a higher likelihood of obstetric complications. Research conducted in the United Mexican States (Mexico) identified chikungunya infection during pregnancy as an independent risk factor for obstetric complications, with an adjusted odds ratio of 1.6.^19^ Smoking, particularly in males, is a recognized risk factor for severe arthralgia during both the acute and chronic phases of CHIKV infection.^20^

While primarily mosquito-borne, evidence for other transmission routes has emerged. A notable finding is the prolonged presence of CHIKV in human semen. Studies have detected infectious virus in semen for up to 56 days post-infection, indicating the potential for sexual transmission of CHIKV and adding another dimension to its epidemiology and control.^21^

## 3 Virology

### 3.1 Virus structure and genome

CHIKV is an alphavirus from the *Togaviridae* family, characterized by a spherical, enveloped structure and a positive single-stranded RNA genome.^22^ The replication of the genome takes place in membranous replication structures known as “spherule”. The 5′ region of the genome encodes four non-structural proteins (nsP1–4), initially synthesized as the polyprotein P1234, assembled into viral replication complex (VRC), which is essential for viral RNA replication.^23^ In the VRC, nsP4 functions as the RNA-dependent RNA polymerase, while nsP2 exhibits helicase activity and cleaves the nsPs from a viral polyprotein precursor to form mature VRC, nsP3 plays a part in bringing host factors to the spherule, nsP1 acts as the membrane anchor for the complex, creating dodecameric pores that connect with the membrane at the spherule necks to control their entry.^24^ Mutations within nsP1 have been shown to attenuate viral virulence.^25^ By suppressing NF-κB activation, nsP3 helps the virus evade early host defenses and establish a foothold in the host.^26^ The nsP4 protein serving as the central catalytic enzyme of the VRC. Mutations in nsP4 can have profound effects on viral replication and can also confer resistance to antiviral drugs, such as the nucleoside analog 4′-fluorouridine.^27^ The nsP2 has emerged as the most promising and intensely studied target for antiviral drug development. A wealth of research has focused on identifying small molecule inhibitors of the nsP2 protease.^28^

**Fig. 2:**
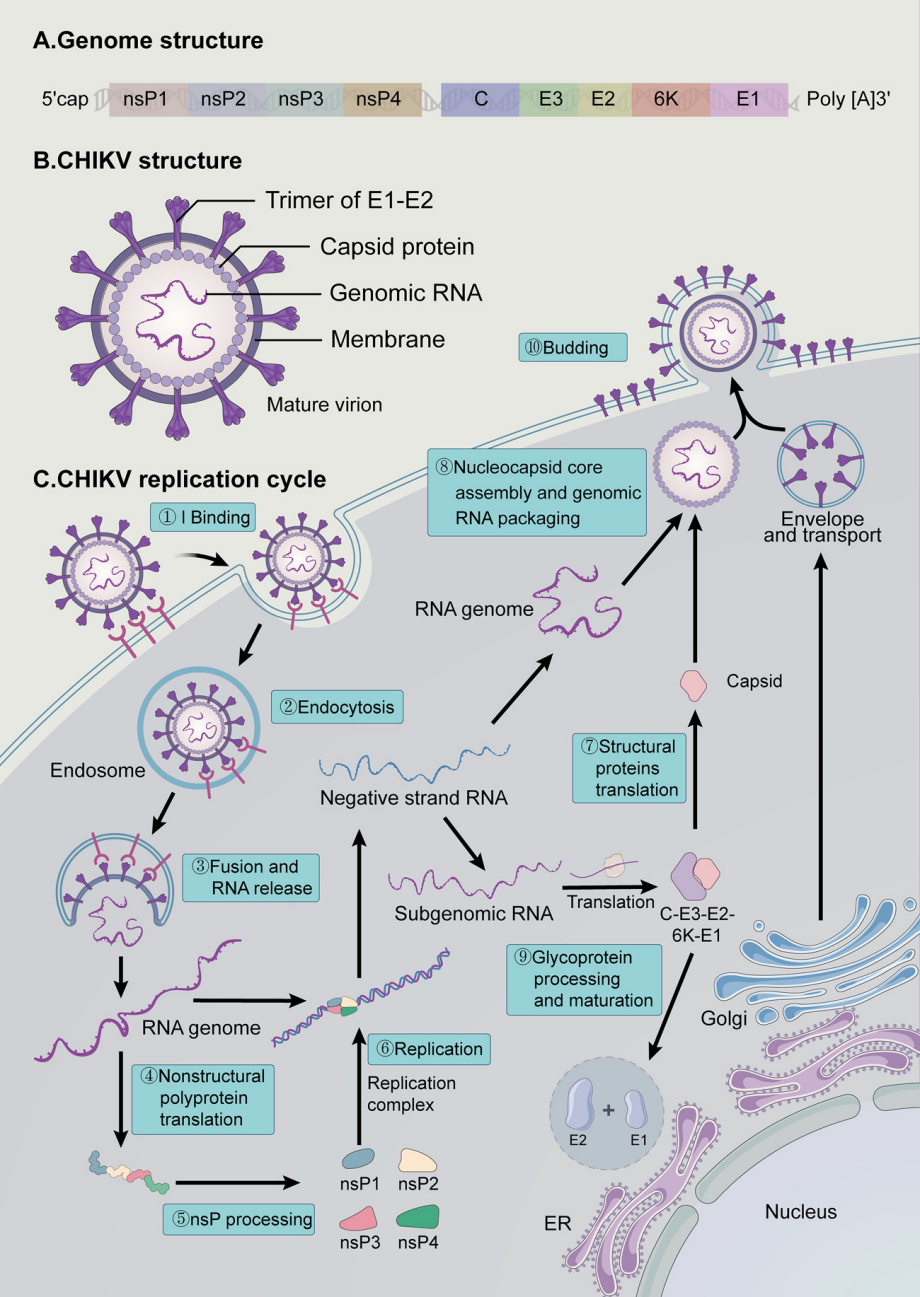
The CHIKV genome organization, virion structure, and viral replication cycle. CHIKV attaches to host receptors (Step 1) and subsequently enters host cells via receptor-mediated endocytosis (Step 2). The acidic environment within endosomes triggers the fusion of the endosomal membrane with the viral envelope, releasing the nucleocapsid into the cytoplasm (Step 3). Disassembly of the nucleocapsid leads to the liberation of the viral genome, which then utilizes the host cell's translational machinery to translate the non-structural polyprotein (nsP1–4) (Step 4). The polyprotein is cleaved into separate nonstructural proteins by viral proteases, predominantly nsP2 (Step 5). Non-structural proteins form VRCs on intracellular membranes and replicate negative-strand RNA (Step 6). Subgenomic RNAs translate polyprotein precursors (C-E3-E2-6K-E1), which is subsequently cleaved into individual structural proteins (Step 7). Capsid proteins assemble with positive-strand RNA to form nucleocapsids (Step 8). E1 and E2 are co-transported into the ER and undergo glycosylation (Step 9). Viral particles are assembled and released by budding through the plasma membrane, where they acquire an envelope embedded with viral glycoproteins (Step 10). The figure was adapted from References 107–108.^107^, ^108^ *Abbreviations*: CHIKV, Chikungunya virus; VRC, viral replication complex; ER, endoplasmic reticulum.

One-third of the genome encodes structural proteins, such as the capsid protein and envelope glycoproteins E1 and E2. Host proteins SPCS3 and eIF3k interact with E1/E2 glycoproteins, regulating the production of new infectious viral particles. This indicates that the virus hijacks specific components of the host's protein synthesis and trafficking machinery to ensure efficient virion morphogenesis (Fig. 2).^29^

### 3.2 Host-virus interactions

The establishment of a successful infection relies on the virus's ability to co-opt a multitude of host cellular factors and pathways. For example, Catenin-α-1 has been demonstrated to be critically important for a productive CHIKV infection. Co-immunoprecipitation shown that Catenin-α-1 can interact with nsP2, and silencing its gene via small interfering RNA (siRNA) can significantly reduce viral particle formation and enhance the survival rate of infected cells.^30^ The host protein BCL2 interacting protein 3 (BNIP3) has been shown to be a key regulator of the early stages of CHIKV replication. BNIP3 regulates CHIKV infection following virus entry and membrane hemifusion. Depletion of BNIP3 increases the expression of CHIKV proteins translated from the viral genomic and subgenomic RNAs. However, this effect is independent of the protein's functions in autophagy and cell death.^31^ The matrix-remodeling associated 8 (MXRA8) protein has been confirmed as a key receptor that mediates the internalization of CHIKV into susceptible cells.^32^ MXRA8 is predominantly expressed in dermal fibroblasts, bone marrow, and synovium.^33^ In post-exposure treatment experiments, administration of anti-MXRA8 monoclonal antibody at 8 or 24 hours post viral inoculation reduced CHIKV infection in the ankles and muscles.^34^

Conversely, host cells also possess intrinsic defense mechanisms that can restrict viral infection. The T-cell immunoglobulin and mucin domain-containing protein 1 has been found to inhibit the release of newly formed CHIKV particles from infected cells.^35^ The host protein nucleophosmin 1 has been identified as an antiviral factor that suppresses CHIKV replication through its role in modulating the expression of interferon-stimulated genes (ISGs).^36^

### 3.3 Viral expansion and adaptation

CHIKV has transitioned from its ancestral sylvatic foci in Sub-Saharan Africa to urban transmission cycles. Phylogenetic research has identified three primary genotypes of CHIKV: West African, Asian, and ECSA. The ECSA genotype strains are linked to higher viremia and more severe symptoms compared to the Asian genotype.^37^

In 2004, CHIKV re-emerged in East Africa and subsequently spread worldwide, leading to epidemics in the Indian Ocean islands, Asia, Europe, and Oceania. The epidemic strains were mainly of the ECSA genotype. In 2013, the Asian genotype strains spread to the Americas, causing widespread outbreaks.^38^ Acquired key mutations enhanced its fitness in the *Aedes albopictus* mosquito vector.^39^ The E1-A226V mutation, which facilitates viral dissemination from the mosquito midgut.^40^ The cumulative effect of these genetic changes has been the transformation of CHIKV into a more efficient and widespread pathogen, capable of causing explosive outbreaks far beyond its historical geographical limits.^41^

## 4 Immune response and pathogenesis

The immune response to CHIKV infection is intricate, involving both innate and adaptive components. The innate immune system serves as the initial defense mechanism against viral infections. Type I interferons (IFNs) are central to this response. Interferon-induced transmembrane proteins (IFITMs), a subset of ISGs, are essential in limiting CHIKV infection. IFITMs, whose expression is triggered by toll-like receptor signaling, inhibit viral entry, illustrating a crucial mechanism of innate immune control over viruses.^42^ In the adaptive immune response, antibodies play a crucial role. IgG enhances the neutralizing ability of human immune sera, with a positive baseline CHIKV plaque reduction neutralization test (PRNT) titer linked to protection against symptomatic CHIKV infection, as observed in a study conducted in Republic of the Philippines.^43^

CHIKV's strategies for immune evasion, with an emphasis on type I IFN responses. The cyclic GMP-AMP synthase (cGAS)-stimulator of interferon genes pathway is crucial for initiating interferon gene expression in response to tissue damage, cellular stress, and infections. CHIKV infection leads to a significant decrease in cGAS expression. CHIKV nsP2 and E1/E2 suppress the activation of the IFNβ-promoter that is triggered by the melanoma differentiation-associated protein 5/retinoic acid-inducible gene I receptor signaling pathway.^44^ The nsP3 protein plays a direct role in suppressing the NF-κB pathway, which is critical for producing pro-inflammatory cytokines and antiviral molecules.^26^ Another key strategy for immune evasion involves the disruption of antigen presentation. The nsP2 disrupts the major histocompatibility complex class I pathway. The virus evades cytotoxic T lymphocyte detection and destruction by inhibiting the presentation of viral antigens on infected cell surfaces, thereby enhancing its persistence and replication.^45^ CHIKV can create stable tunnels between cells, shielding it from neutralizing antibodies and promoting effective intercellular transmission both *in vitro* and *in vivo*.^46^

**Fig. 3:**
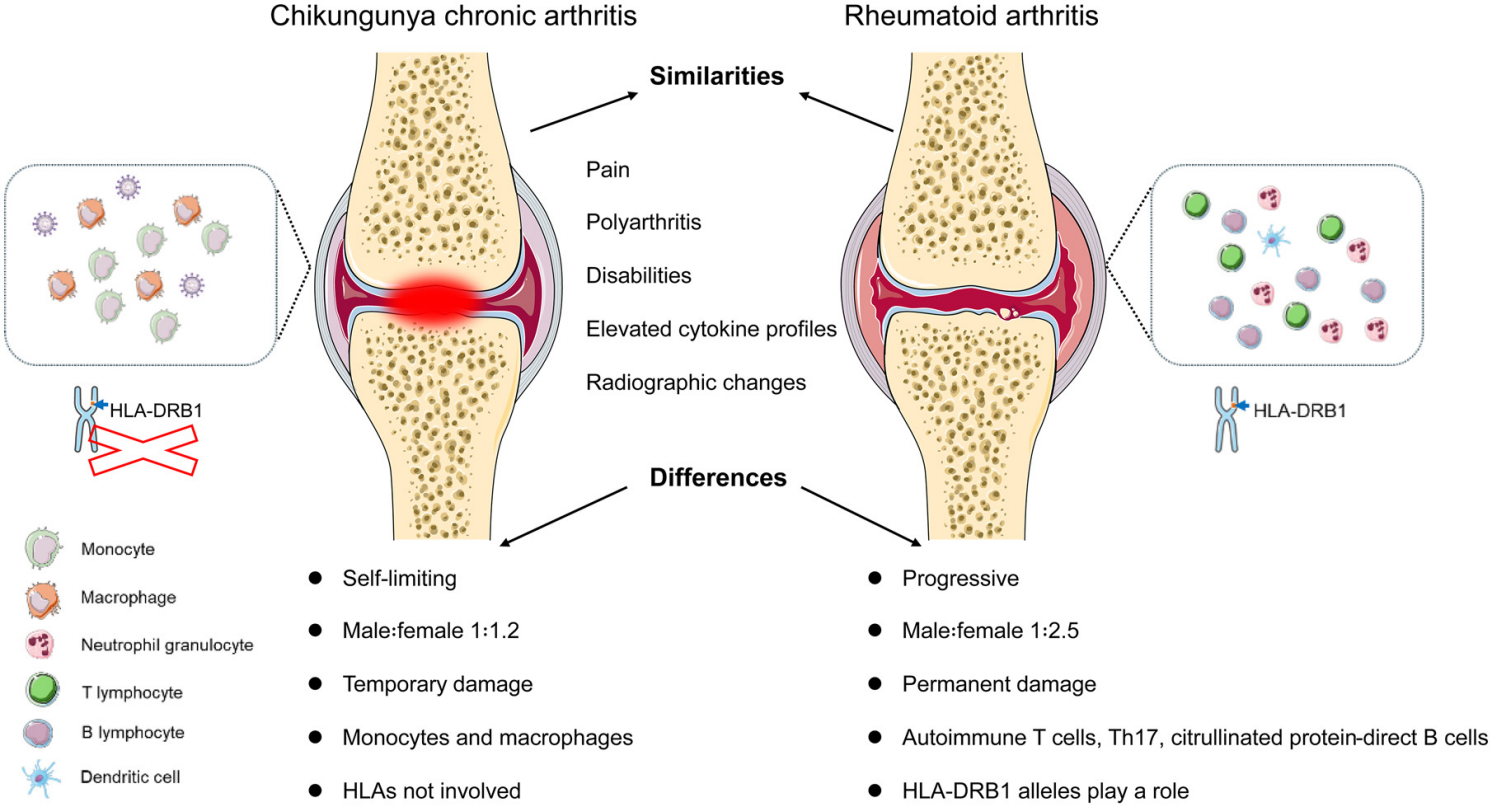
Similarities and differences between chikungunya virus-induced chronic arthritis and rheumatoid arthritis.

The inflammatory response in the joints is driven by a massive infiltration of immune cells. Macrophages expressing high levels of CD64 (FcγRI) are key players in the inflammatory cascade within affected joints.^47^ Although chronic arthritis caused by CHIKV shares similarities with rheumatoid arthritis in terms of clinical manifestations and disease course, the two differ in pathogenesis and prognosis (Fig. 3).^48^ CHIKV infection can induce lasting epigenetic changes in mesenchymal stromal cells, altering their function and promoting a persistent pro-inflammatory state within the joint, even in the absence of replicating virus. ^49^ A marked decrease in regulatory T cells (Tregs) is closely linked to the onset of CHIKV-induced arthritis. The reduction of Tregs probably results in unregulated activation of effector T cells and other immune cells within the joints, promoting chronic inflammation.^50^

Recent studies have confirmed that CHIKV can infect human articular chondrocytes, this infection leads to cell death and the production of inflammatory mediators, contributing directly to cartilage damage and the development of arthritis.^51^ CHIKV infection can also impair the function of osteogenic cells. Bone marrow-derived mesenchymal stem cells and osteogenic cells are susceptible to CHIKV infection. This infection leads to reduced expression of RUNX2, a key regulator of bone cell differentiation. Consequently, the functional abilities of osteogenic cells are impaired, as seen in lower alkaline phosphatase production and diminished matrix mineralization.^52^

The neurological complications of CHIKV, though less common, can be severe. The virus can penetrate the blood-brain barrier and infect central nervous system cells. Experimental model research indicates that CHIKV infection triggers apoptosis in microglia, the brain's resident immune cells. The loss of these crucial cells can disrupt central nervous system homeostasis and trigger neuroinflammation. This pathophysiological process may explain some of the neurological symptoms, such as encephalitis and myelopathy.^53^ The severity of this neurotoxicity appears to be dependent on both the age of the host and the specific strain of the virus, with younger individuals and certain viral genotypes being associated with more severe neurological outcomes.^54^ In rare but devastating cases, CHIKV has been linked to severe outcomes such as acute lower limb flaccid paralysis.^55^

## 5 Clinical manifestations

The acute phase of CHIKV infection usually presents with a sudden high fever exceeding 39°C, accompanied by symptoms such as arthralgia, back pain, headache, nausea, vomiting, arthritis, rash, and conjunctivitis. In a 2011 rural Bangladesh outbreak, the attack rate was 29%, with 76% of confirmed cases experiencing rash alongside fever and joint pain.^56^ A study of patients in the Caribbean region of the Republic of Colombia identified the primary signs and symptoms as lower limb arthralgia (96%), fever (91%), upper limb arthralgia (85%), rash (64%), and headache (57%).^57^

Atypical presentations can also occur, especially in children. In the Republic of Suriname, children with CHIKV infection presented with symptoms such as acute-onset fever, painful knees, rash, hypotension, tachycardia, and meningism.^58^ Ocular complications, including uveitis, have been reported, which may present at the time of systemic manifestations or as a delayed presentation.^59^

Although most CHIKV infections are self-limiting, some patients may develop complications. A study conducted in Martinique and Guadeloupe during the 2013–2014 outbreak reported severe cases, including Guillain-Barré syndrome, encephalitis, and severe sepsis, among patients in the intensive care unit.^60^

Chronic symptoms, such as persistent or relapsing-remitting polyarthralgias, polyarthritis, and myalgias, can endure for months to years, significantly impacting patients' quality of life.^61^ A systematic review and meta-analysis determined that the combined prevalence of post-chikungunya chronic inflammatory rheumatism was 40.22% among 5,702 patients.^62^

The intrapartum vertical transmission of CHIKV from an infected mother to her newborn poses a significant risk. Follow-up studies of children infected during the perinatal period have revealed long-term cognitive and behavioral impairments, indicating that early-life infection can have lasting consequences on neurological function.^63^ Maternal infection with CHIKV and other arboviruses, such as the Zika virus, has been linked to a heightened risk of miscarriage, underscoring the potential dangers during pregnancy.^64^ However, studies in animal models have shown that maternal immunity conferred through breast milk can enhance the resistance of offspring to subsequent CHIKV infection, underscoring the immunological benefits of breastfeeding in endemic areas.^65^ Furthermore, children are not immune to the chronic consequences of the disease. Recent studies have demonstrated that children contracting CHIKV can also experience chronic musculoskeletal symptoms lasting months or years, challenging the previous belief that it was mainly an adult issue. This chronicization of the disease in pediatric populations represents a major, and perhaps under-recognized, public health issue.^66^

## 6 Diagnostics

Differentiating CHIKV infection from other febrile illnesses can be challenging due to the overlapping clinical symptoms. Diseases like dengue fever, Zika virus infection, and rheumatoid arthritis can exhibit similar symptoms, including fever, rash, and joint pain.^67^

During the initial week of illness, when viremia is present, reverse transcription-polymerase chain reaction demonstrates high sensitivity and specificity.^68^ Isothermal methods like reverse transcription loop-mediated isothermal amplification have been created for CHIKV detection. These methods enable quick and sensitive viral RNA detection without the need for advanced thermal cycling equipment, making them ideal for low-resource environments.^69^, ^70^ Furthermore, research has validated the use of alternative sample types for diagnosis. Detecting CHIKV RNA in saliva and urine provides a less invasive alternative to blood draws, beneficial for large-scale screening and pediatric populations.^71^

An evaluation of the VIDAS® automated immunoassay system for detecting anti-CHIKV IgM and IgG antibodies confirmed its utility for routine diagnostics.^72^ The PRNT is another serological method used to detect neutralizing antibodies, crucial for evaluating immunity.^43^

## 7 Prevention and therapy

### 7.1 Prevention strategies

Novel biotechnological approaches are also being explored to combat CHIKV at the vector level. One innovative strategy involves the use of engineered ribozymes—catalytic RNA molecules—designed to specifically target and cleave the CHIKV RNA genome. When expressed in mosquitoes, these ribozymes can inhibit viral replication within the vector, thereby reducing or blocking its transmission to humans. This represents a form of genetic vector control aimed at making mosquito populations resistant to the virus.^73^

Transgenic mosquitoes have been genetically modified to produce anti-CHIKV single-domain antibodies in their midgut. This strategy aims to create a transmission-blocking mosquito population that is incapable of sustaining and transmitting the virus.^74^ Atovaquone, an antimalarial drug, has been shown to inhibit CHIKV transmission in *Aedes aegypti*, indicating its potential for repurposing as a transmission-blocking agent in humans.^75^

The polyclonal antibody has been shown to be effective in preventing CHIKV infection in animal models when administered prophylactically.^76^ It has been demonstrated that the interaction of anti-CHIKV IgG antibodies with the Fc-gamma receptor IIIa on immune cells, such as natural killer cells, significantly enhances their protective efficacy.^77^ Furthermore, understanding the targets of protective antibodies is crucial. The B domain of the E2 envelope protein has been identified as a target for cross-neutralizing antibodies that can recognize multiple alphaviruses.^78^

Socioeconomic factors significantly influence the risk of arboviral infections. Given the increasing severity of deltamethrin resistance in *Aedes albopictus*, it is of great importance to control vectors through comprehensive measures.^79^ Studies have shown that factors such as housing quality, access to piped water, and waste management practices can influence the abundance of mosquito breeding sites and, consequently, the risk of infection to viruses like CHIKV and Dengue.^80^ After the outbreak in Guangdong Province, China in 2025, the health authorities implemented a series of response measures: (1) CHIKV PCR testing was universally conducted among high-risk populations and febrile patients to improve the detection rate of patients; (2) Confirmed cases were isolated in designated hospitals equipped with mosquito-proof facilities, including insecticide-treated window screens and bed nets; (3) Comprehensive vector management measures were implemented, encompassing targeted insecticide spraying, elimination of breeding sites, application of biological control, and real-time vector monitoring. These measures led to the timely containment of the outbreak.^81^ These findings highlight that disease control cannot rely solely on biomedical interventions but must also incorporate improvements in social and environmental conditions.

### 7.2 Vaccine development

Different vaccine platforms have been investigated to prevent CHIKV infection, such as inactivation, live-attenuated strains, virus-like particles (VLPs), viral vectors, and mRNA technologies. In the United States, two chikungunya vaccines have received licensing and approval: IXCHIQ (VLA1553), a live-attenuated vaccine^82^, and VIMKUNYA (PXVX0317), a VLP vaccine.^83^ A crucial phase 2 trial of the VIMKUNYA vaccine confirmed its safety and ability to provoke an immune response in adults without prior CHIKV exposure, showing a lasting serum neutralizing antibody response for up to two years after vaccination.^84^ This trial also highlighted the vaccine's favorable safety profile, with no serious adverse events reported, which is consistent with findings from other studies on VLP-based vaccines. The VIMKUNYA vaccine demonstrated consistent immunogenicity across various age groups in a phase 3 trial with participants aged 12–64 years. The trial demonstrated a strong and swift immune response, with a seroresponse rate of 97.8% in the vaccine group, significantly higher than the 1.2% observed in the placebo group.^85^ Another phase 3 trial has confirmed the safety and efficacy of the VIMKUNYA vaccine in adults aged 65 and older, a group at higher risk for severe CHIKF complications. The trial showed that the vaccine was well-tolerated, offering significant protection within two weeks after administration, with no serious adverse events or deaths linked to the vaccine.^86^

BBV87, a live-attenuated vaccine, has demonstrated safety and efficacy in protecting non-human primates from CHIKV infection, endorsing its continued clinical development.^87^ For specific populations, such as travelers, inactivated vaccines are also being developed, which may offer a different safety profile compared to live-attenuated versions.^88^

A significant advancement has been the development of a vaccine candidate based on E2-B nanoparticles. These nanoparticles display a portion of the CHIKV E2 glycoprotein and have been shown to induce potent and broadly neutralizing antibody responses in preclinical models.^89^ A novel vaccine candidate using a non-replicating, attenuated CHIKV RNA packaged within liposomes has been developed. When administered to mice, this liposome-formulated RNA vaccine induced potent protective immunity against a lethal CHIKV challenge.^90^ Other candidate vaccines with available clinical trial results are shown in Table 1.

**Table 1:** An overview of the latest CHIKV vaccine candidates available clinical trial results (except licensed vaccine)^91^.

Platform	Vaccine	Stage	Enrollment	Target Population	ID^a^	Last update post	Mean antibody titer (95% CI or mean ± SD)
Viral vectors	MV-CHIK-204	Phase II	34	Healthy adults in previously epidemic area	NCT03101111	2021-07-19	1,280 (920.5–1,779.9) in baseline seropositive cohort on day 56
Viral vectors	MV-CHIK-202	Phase II	263	Healthy adults	NCT02861586	2021-10-29	174.8 ± 436.11 in high dose group on day 56, 100% seroconversion after the second vaccination
VLP	VRC-CHKVLP059-00-VP	Phase I	25	Healthy adults	NCT01489358	2016-07-25	5,390 (1,865–15,573) in 24 weeks
VLP	VRC-CHKVLP059-00-VP	Phase II	400	Healthy adults	NCT02562482	2020-10-22	2,004.5 (1,680.1–2,391.5) on day 56
Viral vectors	V184-005	Phase II	60	Healthy adults	NCT03635086	2021-10-22	45.7 (21.6–97.0) in high dose group on day 56, 100% seroconversion after the second vaccination
Viral vectors	V184-006	Phase II	41	Previously exposed adults	NCT03807843	2022-09-08	1.9 (1.5–2.3) on day 56
mRNA-based vaccine	VAL-181388	Phase I	60	Healthy adults in a non-endemic Chikungunya region	NCT03325075	2024-06-10	92.8 (43.6–197.6) on day 56

*Abbreviations:* CI, confidence interval; SD, standard deviation; VLP, virus-like particle vaccine.

a ID of ClinicalTrials.gov, data available: https://clinicaltrials.gov/.

### 7.3 Investigational antiviral agents

Numerous investigational antiviral agents have been studied for their potential against CHIKV. As discussed, the nsP2 protease is a prime target. Synthetic small molecules like acrylamide derivatives have been developed to specifically block its function.^92^ The nsP3 protein, with its role in VRC assembly, has also been targeted. The antiviral effectiveness of Ag/NiO and Ag_2_O/NiO/ZnO mixed metal oxide nanocomposites against CHIKV was assessed using *in vitro* tests., by disrupting function of nsP3 and inhibiting viral replication, this presents high engineering potential for broad-spectrum antiviral activity.^93^ Beyond targeting individual viral proteins, researchers are exploring compounds with broader mechanisms of action. Research indicates that L-cysteine can inhibit CHIKV infection *in vitro*, implying that altering cellular metabolic or redox states may serve as an effective antiviral strategy.^94^

The search for novel chemical scaffolds has also been fruitful. Benzothiazole (BTA) derivatives can reduce viral replication by 98%. Molecular docking indicated that there are strong binding affinities to CHIKV's non-structural proteins and envelope glycoproteins. Infrared spectroscopy confirmed it's interaction with the glycoprotein complex and lipids, highlight BTA derivatives as promising CHIKV inhibitors.^95^ Mechanistic tests showed that sulfur- and selenium-containing benzotriazoles disrupted viral adsorption, had virucidal properties, and blocked several stages of the replication cycle, emphasize these compounds as potential dual-action antiviral agents with wide-ranging effects against Zika virus (ZIKV) and CHIKV.^96^ Thiocyanate compounds^97^ and chlorinated biscoumarin derivatives^98^ have been identified as potent inhibitors of CHIKV replication in cell culture models. Additional preclinical studies are needed to assess their therapeutic potential.

Drug repurposing offers a faster path to clinical use. For instance, the HIV reverse transcriptase inhibitor Efavirenz has been shown to possess anti-CHIKV activity.^99^ Similarly, certain drugs approved for treating hepatitis C virus have demonstrated inhibitory effects against CHIKV, suggesting they could be repurposed for this new indication.^100^ Etravirine, an approved non-nucleoside reverse transcriptase inhibitor for HIV treatment, exhibits inhibitory effects on West Nile Virus and CHIKV, indicating its potential as a broad-spectrum antiviral agent.^101^ Nifuroxazide, an oral nitrofuran antibiotic, exhibits broad-spectrum antiviral activity by inhibiting the replication of various insect-borne viruses, including CHIKV.^102^ Furthermore, derivatives of artesunate, an antimalarial drug, such as Anthrone-Spirolactam, have been synthesized and found to have potent anti-CHIKV activity.^103^

RNA interference offers a highly specific way to silence viral genes. A novel system using zeolitic imidazolate framework-coated carbon nanodots (ZIF-C) has been developed to effectively deliver siRNA molecules into infected cells. ZIF-C confers maximal protection to the loaded nucleic acids against nuclease-mediated degradation. By targeting the E2 and nsP1 genes, viral replication and infectivity are reduced.^104^ Peptide-based inhibitors represent another promising class of biologics. A hybrid peptide named GA-Hecate was designed and shown to have potent inhibitory activity against CHIKV.^105^ Similarly, certain dipeptides have been identified that exhibit antiviral activity against both CHIKV and Zika virus, suggesting they may target a conserved process in flavivirus and alphavirus replication.^106^

## 8 Conclusion

Due to the long-term and severe consequences brought about by the CHIKV infection, CHIKF has clearly become a persistent and highly threatening global health issue. There is an urgent need for the development of novel molecular and antigen detection assays with high sensitivity, rapid recognition, accessibility. Future fundamental research on CHIKV should prioritize elucidating its genetic variation, host-pathogen interactions, and mechanisms of vector adaptation. In the development of antiviral agents, significant challenges include the virus's capacity to mutate and develop resistance to antiviral therapies. Therefore, establishing efficient antiviral screening platforms for drug discovery is imperative. Promising targets for breakthroughs include the inhibition of the capping, macrodomain, and capsid protease functions of nsPs. Other therapeutic strategies under investigation involve targeting host cell pathways that either facilitate or inhibit viral replication, such as fatty acid synthesis and cholesterol trafficking pathways; dysregulating endosome acidification to inhibit viral entry; inhibiting nucleobase biosynthesis; or using immunomodulatory therapies to stimulate an interferon response. Given the absence of specific treatments for CHIKV, the developing protective monoclonal antibody therapy may temporarily address the disease's deterioration.

In vaccine development, it is imperative to ensure long-term protection and safety, particularly among high-risk populations such as children, the elderly, and individuals with comorbidities. A comprehensive understanding of humoral immunity and a detailed examination of antibody-dependent enhancement mechanisms are critical for the design of effective and safe vaccines. An optimal vaccine should possess thermostability and be facile in production, transportation, and storage, especially in low- and middle-income countries that are disproportionately affected by CHIKV.

Collectively, the recent advances in virology, immunology, clinical medicine, and drug development provide a solid and expanding foundation of knowledge and tools to mitigate the impact of CHIKV on human populations worldwide. The advancement of this field necessitates interdisciplinary research.

## CRediT authorship contribution statement

**Xin Zhang:** Writing – review & editing, Writing – original draft, Funding acquisition. **Xiaoxi Li:** Writing – review & editing, Writing – original draft. **Tianjun Jiang:** Writing – review & editing, Writing – original draft, Supervision, Funding acquisition. **Junliang Fu:** Writing – review & editing, Writing – original draft, Supervision, Project administration, Funding acquisition.

## Informed consent

Not applicable.

## Organ donation

Not applicable.

## Ethical statement

Not applicable.

## Data availability statement

Data availability is not applicable to this review as no new data were created or analyzed in this study.

## Animal treatment

Not applicable.

## Generative AI

During the preparation of this work the authors used MediPen (https://www.home-for-researchers.com/#/medipen) and Doubao (https://www.doubao.com/chat) for grammar correction and stylistic improvements. After using this tool, the authors reviewed and edited the content and take full responsibility for the content of the published article.

## Funding

This study was supported in part by grants from the National Key R&D Program of China (2022YFC2305004 and 2023YFC2308100).

## Declaration of competing interest

The authors declare that they have no known competing financial interests or personal relationships that could have appeared to influence the work reported in this paper.

## References

[1] Acosta-Reyes J., Tuesca R., Navarro-Lechuga E. (2025). Chronicity and quality of life in Chikungunya virus infection: a cross-sectional study in Barranquilla, Colombia. J Microbiol Immunol Infect.

[2] Feng Y., Chang F.F., Yang Y. (2025). From dengue to chikungunya: Guangdong as a sentinel for arboviral threats in East Asia. Biosci Trends.

[3] Disease Outbreak News; Chikungunya virus disease- Global situation. World Health Organization, 2025. Available from: https://www.who.int/emergencies/disease-outbreak-news/item/2025-DON581.

[4] de Souza W.M., Gaye A., Ndiaye E.H. (2024). Serosurvey of chikungunya virus in old world fruit bats, Senegal, 2020–2022. Emerg Infect Dis.

[5] Li N., Peng C.C., Yuan Y.G. (2023). A new cluster of chikungunya virus West Africa genotype isolated from *Aedes albopictus* in China. J Infect.

[6] Allen S.W., Ribeiro Dos Santos G., Paul K.K. (2024). Results of a nationally representative seroprevalence survey of chikungunya virus in Bangladesh. J Infect Dis.

[7] Anjos R.O., Portilho M.M., Jacob-Nascimento L.C. (2023). Dynamics of chikungunya virus transmission in the first year after its introduction in Brazil: a cohort study in an urban community. PLoS Negl Trop Dis.

[8] Giovanetti M., Vazquez C., Lima M. (2023). Rapid epidemic expansion of chikungunya virus east/central/south African lineage, Paraguay. Emerg Infect Dis.

[9] Parker D.M., Haileselassie W., Hailemariam T.S. (2025). High seroprevalence of antibodies to Dengue, Chikungunya, and Zika viruses in Dire *Dawa*, Ethiopia: a cross-sectional survey in 2024. PLoS Negl Trop Dis.

[10] Visser T.M., Wang H.D., Abbo S.R. (2025). Effect of chikungunya, Mayaro and *Una virus* coinfection on vector competence of *Aedes aegypti* mosquitoes. One Health.

[11] Wesselmann K.M., Baronti C., Nougairède A. (2025). Development and evaluation of a duplex RT-qPCR assay for the detection and identification of Mayaro and chikungunya viruses. J Clin Microbiol.

[12] Tinto B., Bicaba B., Kagoné T.S. (2024). Co-circulation of two Alphaviruses in Burkina Faso: Chikungunya and O’nyong nyong viruses. PLoS Negl Trop Dis.

[13] Wesselmann K.M., Luciani L., Thirion L. (2024). Analytical and clinical evaluation of a duplex RT-qPCR assay for the detection and identification of o’nyong-nyong and chikungunya virus. Emerg Microbes Infect.

[14] Azman I.K., Chan Y.F., Chua C.L. (2024). A change in circulating chikungunya virus variant impacts *Aedes aegypti* vector competence and spatiotemporal distribution of disease in Malaysia. PLoS Negl Trop Dis.

[15] Mehta D., Chaudhary S., Sunil S. (2024). Oxidative stress governs mosquito innate immune signalling to reduce chikungunya virus infection in *Aedes*-derived cells. J Gen Virol.

[16] Bernal-Valle S., Monteiro de Mello Mares-Guia M.A., Vieira Santos de Abreu F. (2025). Natural exposure to Chikungunya virus in golden-headed lion tamarin (*Leontopithecus chrysomelas*, Kuhl, 1820) from non-protected areas in southern *Bahia*, Brazil: Implications and significance. PLoS Negl Trop Dis.

[17] Aranda-Coello J.M., Machain-Williams C., Weber M. (2025). Serologic surveillance for orthoflaviviruses and chikungunya virus in bats and opossums in Chiapas, Mexico. Viruses.

[18] Micheleto J.P.C., Melo K.A., Veloso F.C.S. (2025). Risk factors for mortality in patients with chikungunya: a systematic review and meta-analysis. Trop Med Int Health.

[19] de Jesús, Ortiz-Mesina J., Caballero-Hoyos J.R., Trujillo X. (2019). Obstetric complications of dengue and chikungunya in the pregnant patient: case-control study. Rev Med Inst Mex Seguro Soc.

[20] Delgado-Enciso I., Paz-Michel B., Melnikov V. (2018). Smoking and female sex as key risk factors associated with severe arthralgia in acute and chronic phases of Chikungunya virus infection. Exp Ther Med.

[21] Martins E.B., de Bruycker-Nogueira F., Rodrigues C.D.S. (2022). Chikungunya virus shedding in *Semen*: a case series. Viruses.

[22] Manzoor K.N., Javed F., Ejaz M. (2022). The global emergence of Chikungunya infection: an integrated view. Rev Med Virol.

[23] Pietilä M.K., Hellström K., Ahola T. (2017). Alphavirus polymerase and RNA replication. Virus Res.

[24] Jones R., Hons M., Rabah N. (2023). Structural basis and dynamics of Chikungunya alphavirus RNA capping by nsP1 capping pores. Proc Natl Acad Sci USA.

[25] Chamberlain J., Dowall S.D., Smith J. (2025). Attenuation of chikungunya virus by a single amino acid substitution in the nsP1 component of a non-structural polyprotein. Viruses.

[26] Roberts G.C., Stonehouse N.J., Harris M. (2025). The chikungunya virus nsP3 macro domain inhibits activation of the NF-κB pathway. Viruses.

[27] Yin P.Q., Sobolik E.B., May N.A. (2025). Mutations in chikungunya virus nsP4 decrease viral fitness and sensitivity to the broad-spectrum antiviral 4’-Fluorouridine. PLoS Pathog.

[28] Ghoshal A., Tse E.G., Hossain M.A. (2025). A covalent chemical probe for Chikungunya nsP2 cysteine protease with antialphaviral activity and proteome-wide selectivity. Sci Rep.

[29] Yao Z.L., Ramachandran S., Huang S. (2024). Interaction of chikungunya virus glycoproteins with macrophage factors controls virion production. EMBO J.

[30] Chatterjee S., Subudhi B.B., Chattopadhyay S. (2023). A hidden gem Catenin-α-1 is essential for Chikungunya virus infection. Microbiol Spectr.

[31] Echavarria-Consuegra L., Dinesh Kumar N., van der Laan M. (2023). Mitochondrial protein BNIP3 regulates Chikungunya virus replication in the early stages of infection. PLoS Negl Trop Dis.

[32] Feng F., Bouma E.M., Hu G.W. (2023). Colocalization of chikungunya virus with its receptor MXRA8 during cell attachment, internalization, and membrane fusion. J Virol.

[33] Zhang R., Earnest J.T., Kim A.S. (2019). Expression of the Mxra8 receptor promotes alphavirus infection and pathogenesis in mice and *Drosophila*. Cell Rep.

[34] Zhang R., Kim A.S., Fox J.M. (2018). Mxra8 is a receptor for multiple arthritogenic alphaviruses. Nature.

[35] Reyes Ballista J.M., Hoover A.J., Noble J.T. (2024). Chikungunya virus release is reduced by TIM-1 receptors through binding of envelope phosphatidylserine. J Virol.

[36] Pradeep P., Sivakumar K.C., Sreekumar E. (2023). Host factor nucleophosmin 1 (NPM1/B23) exerts antiviral effects against chikungunya virus by its interaction with viral nonstructural protein 3. Microbiol Spectr.

[37] Lau J.Z.H., Chua C.L., Chan Y.F. (2023). Replication and innate immune responses of two chikungunya virus genotypes in human monocyte-derived macrophages. J Gen Virol.

[38] Sam I.C., Kümmerer B.M., Chan Y.F. (2015). Updates on chikungunya epidemiology, clinical disease, and diagnostics. Vector Borne Zoonotic Dis.

[39] Frumence E., Piorkowski G., Traversier N. (2025). Genomic insights into the re-emergence of chikungunya virus on Réunion Island, France, 2024 to 2025. Eurosurveillance.

[40] Romano G., Pavesi A., Ferrari A. (2025). Chikungunya cases with vector-adaptive mutations detected in Italy imported from Madagascar. J Travel Med.

[41] Liu Q., Shen H., Gu L. (2025). Chikungunya virus in Europe: a retrospective epidemiology study from 2007 to 2023. PLoS Negl Trop Dis.

[42] Gumpangseth N., Villarroel P.M.S., Diack A. (2025). IFITMs exhibit antiviral activity against Chikungunya and Zika virus infection *via* the alteration of TLRs and RLRs signaling pathways. Sci Rep.

[43] Yoon I.K., Alera M.T., Lago C.B. (2015). High rate of subclinical chikungunya virus infection and association of neutralizing antibody with protection in a prospective cohort in the Philippines. PLoS Negl Trop Dis.

[44] de Oliveira Souza R., Duarte J.W.B., Della Casa V.S. (2024). Unraveling the complex interplay: immunopathology and immune evasion strategies of alphaviruses with emphasis on neurological implications. Front Cell Infect Microbiol.

[45] Ware B.C., Parks M.G., da Silva M.O.L. (2024). Chikungunya virus infection disrupts MHC-I antigen presentation *via* nonstructural protein 2. PLoS Pathog.

[46] Yin P.Q., Davenport B.J., Wan J.J. (2023). Chikungunya virus cell-to-cell transmission is mediated by intercellular extensions *in vitro* and *in vivo*. Nat Microbiol.

[47] Lum F.M., Chan Y.H., Teo T.H. (2024). Crosstalk between CD64^+^MHCII+ macrophages and CD4^+^ T cells drives joint pathology during chikungunya. EMBO Mol Med.

[48] Kumar R., Ahmed S., Ahmad Parray H. (2021). Chikungunya and arthritis: an overview. Travel Med Infect Dis.

[49] Amaral J.K., Bingham C.O., Taylor P.C. (2023). Pathogenesis of chronic chikungunya arthritis: Resemblances and links with rheumatoid arthritis. Travel Med Infect Dis.

[50] Dobbs J.E., Tritsch S.R., Encinales L. (2022). Regulatory T-cells and GARP expression are decreased in exercise-associated chikungunya viral arthritis flares. Front Immunol.

[51] Legros V., Belarbi E. (2025). Use of recombinant chikungunya virus expressing nanoluciferase to identify chondrocytes as target cells in an immunocompetent mouse model. J Infect Dis.

[52] Roy E., Shi W., Duan B. (2020). Chikungunya virus infection impairs the function of osteogenic cells. mSphere.

[53] Kumar N., Santhoshkumar R., Venkataswamy MM. (2024). Chikungunya virus infection in human microglial C20 cells induces mitochondria-mediated apoptosis. Front Cell Infect Microbiol.

[54] Anderson E.J., Knight A.C., Heise M.T. (2023). Effect of viral strain and host age on clinical disease and viral replication in immunocompetent mouse models of chikungunya encephalomyelitis. Viruses.

[55] Matungala-Pafubel M., Bulabula-Penge J., Matondo-Kuamfumu M. (2024). Lower limb paralysis associated with chikungunya in Kinshasa, the democratic republic of the Congo: survey report. Pathogens.

[56] Khatun S., Chakraborty A., Rahman M. (2015). An Outbreak of Chikungunya in Rural Bangladesh, 2011. PLoS Negl Trop Dis.

[57] Mattar S., Miranda J., Pinzon H. (2015). Outbreak of Chikungunya virus in the north Caribbean area of Colombia: clinical presentation and phylogenetic analysis. J Infect Dev Ctries.

[58] Mac Donald-Ottevanger M.S., Gravenberch-Ramnandanlall C.I., Zijlmans C.W. (2015). [Chikungunya in children]. Ned Tijdschr Geneeskd.

[59] Mahendradas P., Patil A., Kawali A. (2024). Systemic and ophthalmic manifestations of chikungunya fever. Ocul Immunol Inflamm.

[60] Crosby L., Perreau C., Madeux B. (2016). Severe manifestations of chikungunya virus in critically ill patients during the 2013-2014 Caribbean outbreak. Int J Infect Dis.

[61] Arroyo-Ávila M., Vilá LM. (2015). Rheumatic manifestations in patients with chikungunya infection. P R Health Sci J.

[62] Rodríguez-Morales A.J., Cardona-Ospina J.A., Fernanda Urbano-Garzón S. (2016). Prevalence of post-chikungunya infection chronic inflammatory arthritis: a systematic review and meta-analysis. Arthritis Care Res.

[63] Sarton R., Carbonnier M., Robin S. (2025). Perinatal mother-to-child chikungunya virus infection: screening of cognitive and learning difficulties in a follow-up study of the chimere cohort on Reunion Island. Viruses.

[64] de Carvalho A.K.P., Cruz A.C.R., Quaresma J.A.S. (2025). Impact of zika and chikungunya viruses on spontaneous abortions: insights from a reference maternity hospital. Microorganisms.

[65] de Paula Souza J., Santos de Jesus B.L., Giusti A.L. (2023). Breastfeeding by chikungunya virus-infected dams confers resistance to challenge in the offspring. Transl Res.

[66] de Jesus, Pereira B., Brasil M.Q.A., de Jesus, Silva J. (2025). Chikungunya in a pediatric cohort: Asymptomatic infection, seroconversion, and chronicity rates. PLoS Negl Trop Dis.

[67] Miner J.J., Aw-Yeang H.X., Fox J.M. (2015). Chikungunya viral arthritis in the United States: a mimic of seronegative rheumatoid arthritis. Arthritis Rheumatol.

[68] Sajith A., Iyengar V., Varamballi P. (2025). Diagnostic utility of real-time RT-PCR for chikungunya virus detection in the acute phase of infection: a retrospective study. Ann Med..

[69] Wu X.L., Liu G.W., Chang Y.C. (2024). Rapid and sensitive detection of chikungunya virus using one-tube, reverse transcription, semi-nested multi-enzyme isothermal rapid amplification, and lateral flow dipstick assays. J Clin Microbiol.

[70] da Silva S.J.R., de Magalhães J.J.F., Matthews Q. (2024). Development and field validation of a reverse transcription loop-mediated isothermal amplification assay (RT-LAMP) for the rapid detection of chikungunya virus in patient and mosquito samples. Clin Microbiol Infect.

[71] Jacob-Nascimento LC, Portilho MM, Anjos RO, et al. Detection of chikungunya virus RNA in oral fluid and urine: an alternative approach to diagnosis *Viruses*. 2024, 16(2): 235. doi:10.3390/v16020235. 10.3390/v16020235 PMC10891727 38400011

[72] Pereira G.M., Manuli E.R., Coulon L. (2023). Performance evaluation of VIDAS® diagnostic assays detecting anti-chikungunya virus IgM and IgG antibodies: an international study. Diagnostics.

[73] Mishra P., Balaraman V., Fraser M.J. (2023). Maxizyme-mediated suppression of chikungunya virus replication and transmission in transgenic *Aedes aegypti* mosquitoes. Front Microbiol.

[74] Webb E.M., Compton A., Rai P. (2023). Expression of anti-chikungunya single-domain antibodies in transgenic *Aedes aegypti* reduces vector competence for chikungunya virus and Mayaro virus. Front Microbiol.

[75] Wang L.J., Sanon A., Khoiriyah Z. (2023). Tarsal exposure to atovaquone inhibits chikungunya virus transmission by *Aedes aegypti* mosquitoes, but not the transmission of Zika virus. Antiviral Res.

[76] Barker D., Han X.B., Wang E.Y. (2023). Equine polyclonal antibodies prevent acute chikungunya virus infection in mice. Viruses.

[77] Fox J.M., Roy V., Gunn B.M. (2023). Enhancing the therapeutic activity of hyperimmune IgG against chikungunya virus using FcγRIIIa affinity chromatography. Front Immunol.

[78] Powers J.M., Lyski Z.L., Weber W.C. (2023). Infection with chikungunya virus confers heterotypic cross-neutralizing antibodies and memory B-cells against other arthritogenic alphaviruses predominantly through the B domain of the E2 glycoprotein. PLoS Negl Trop Dis.

[79] Mohapatra R.K., Bhattacharjee P., Desai D.N. (2024). Global health concern on the rising dengue and chikungunya cases in the American regions: Countermeasures and preparedness. Health Sci Rep.

[80] Tariq A., Khan A., Mutuku F. (2024). Understanding the factors contributing to dengue virus and chikungunya virus seropositivity and seroconversion among children in Kenya. PLoS Negl Trop Dis.

[81] Tee K.K., Mu D.K., Xia XS. (2025). Explosive chikungunya virus outbreak in China. Int J Infect Dis.

[82] Maurer G., Buerger V., Larcher-Senn J. (2025). Comprehensive assessment of reactogenicity and safety of the live-attenuated chikungunya vaccine (IXCHIQ®). Vaccines.

[83] Raju S., Adams L.J., Earnest J.T. (2023). A chikungunya virus-like particle vaccine induces broadly neutralizing and protective antibodies against alphaviruses in humans. Sci Transl Med.

[84] Bennett S.R., McCarty J.M., Ramanathan R. (2022). Safety and immunogenicity of PXVX0317, an aluminium hydroxide-adjuvanted chikungunya virus-like particle vaccine: a randomised, double-blind, parallel-group, phase 2 trial. Lancet Infect Dis.

[85] Richardson J.S., Anderson D.M., Mendy J. (2025). Chikungunya virus virus-like particle vaccine safety and immunogenicity in adolescents and adults in the USA: a phase 3, randomised, double-blind, placebo-controlled trial. Lancet.

[86] Tindale L.C., Richardson J.S., Anderson D.M. (2025). Chikungunya virus virus-like particle vaccine safety and immunogenicity in adults older than 65 years: a phase 3, randomised, double-blind, placebo-controlled trial. Lancet.

[87] Kempster S.L., Ferguson D., Ham C. (2025). Inactivated viral vaccine BBV87 protects against chikungunya virus challenge in a non-human primate model. Viruses.

[88] Freedman DO. (2025). A new non-live chikungunya vaccine for travellers. J Travel Med.

[89] Tong K.R., Hernandez E.M., Basore K. (2024). Chikungunya virus E2 B domain nanoparticle immunogen elicits homotypic neutralizing antibody in mice. Vaccine.

[90] Rao S., Abeyratne E., Freitas J.R. (2023). A booster regime of liposome-delivered live-attenuated CHIKV vaccine RNA genome protects against chikungunya virus disease in mice. Vaccine.

[91] U.S. Department of Health and Human Services, National Institutes of Health, National Library of Medicine, and National Center for Biotechnology Information, 2025. Available from: https://clinicaltrials.gov/searchcond=chikungunya&term=vaccine.

[92] de Souza B.G., Choudhary S., Vilela G.G. (2023). Design, synthesis, antiviral evaluation, and in silico studies of acrylamides targeting nsP2 from Chikungunya virus. Eur J Med Chem.

[93] Bhatia P., Singh V.A., Rani R. (2023). Cellular uptake of metal oxide-based nanocomposites and targeting of chikungunya virus replication protein nsP3. J Trace Elem Med Biol.

[94] Kumar A., Shrinet J., Sunil S. (2023). Chikungunya virus infection in *Aedes aegypti* is modulated by L-cysteine, taurine, hypotaurine and glutathione metabolism. PLoS Negl Trop Dis.

[95] Feferbaum-Leite S., Marques Cassani N., Aquino Ruiz U.E. (2025). Benzothiazole derivatives as inhibitors of chikungunya virus replicative cycle. Future Med Chem..

[96] Gomes L.S., CC Cirne-Santos (2025). Sulfur/selenium-functionalized benzotriazoles as multifunctional antivirals targeting Zika & Chikungunya. Future Med Chem.

[97] Alagarasu K., Dhote R., Bagad P.K. (2025). Effectiveness of 3-amino-2-thiocyanato-α, β-unsaturated carbonyl compounds against chikungunya virus. Future Med Chem..

[98] Orji C.N., Loeanurit N., Pham V.C. (2025). Chlorinated biscoumarins inhibit chikungunya virus replication in cell-based and animal models. Emerg Microbes Infect.

[99] Nehul S., Rani R., Walia P. (2025). Repurposing efavirenz, the HIV antiretroviral drug for chikungunya virus infection. ACS Infect Dis.

[100] Kalam N., Ali R., Balasubramaniam VR. (2025). Exploring the potential of direct-acting antivirals against Chikungunya virus through structure-based drug repositioning and molecular dynamic simulations. Comput Biol Med.

[101] Zheng X., He Y.H., Xia B.H. (2024). Etravirine prevents west Nile virus and chikungunya virus infection both *in vitro* and *in vivo* by inhibiting viral replication. Pharmaceutics.

[102] Liu Y.G., Xu M.X., Xia B.H. (2024). Nifuroxazide prevents chikungunya virus infection both *in vitro* and *in vivo via* suppressing viral replication. Viruses.

[103] Darole R.S., Bagad P.K., Gonnade R.G. (2023). Synthesis of novel rhodamine type Anthrone Spiro-lactam (ASL) analogues and evaluation of antiviral activity against dengue and chikungunya viruses. Eur J Med Chem.

[104] Tagore R., Alagarasu K., Patil P. (2022). Targeted *in vitro* gene silencing of E2 and nsP1 genes of chikungunya virus by biocompatible zeolitic imidazolate framework. Front Bioeng Biotechnol.

[105] Ayusso G.M., da Silva, Sanches P.R., Carvalho T. (2023). The synthetic peptide GA-*Hecate* and its analogs inhibit multiple steps of the chikungunya virus infection cycle *in vitro*. Pharmaceuticals.

[106] Ayusso G.M., Lima M.L.D., da Silva Sanches P.R. (2023). The dimeric peptide (KKYRYHLKPF)_2_K shows broad-spectrum antiviral activity by inhibiting different steps of chikungunya and zika virus infection. Viruses.

[107] Wang T.Y., Sun Y., Tang YD. (2025). Re-emergence of chikungunya virus in China by 2025: What we know and what to do. PLoS Pathog.

[108] Freppel W., Silva L.A., Stapleford K.A. (2024). Pathogenicity and virulence of chikungunya virus. Virulence.

